# Fine-scale population genetic structure of arctic foxes (*Vulpes lagopus*) in the High Arctic

**DOI:** 10.1186/s13104-017-3002-1

**Published:** 2017-12-01

**Authors:** Sandra Lai, Adrien Quiles, Josie Lambourdière, Dominique Berteaux, Aude Lalis

**Affiliations:** 1 Canada Research Chair on Northern Biodiversity, Centre for Northern Studies and Quebec Center for Biodiversity Science, Université du Québec à Rimouski, 300 Allée des Ursulines, Rimouski, QC G5L 3A1 Canada; 2 UMR7205 ISYEB CNRS-MNHN-EPHE-UPMC, Muséum National d’Histoire Naturelle, CP 51, 75231 Paris Cedex 05, France; 3 UMS 2700 OMSI Service de Systématique Moléculaire, Muséum National d’Histoire Naturelle, CP 26, 75231 Paris Cedex 05, France

**Keywords:** *Vulpes lagopus*, Microsatellite multiplex PCR, Population genetics, Fine-scale genetic structure, Bylot Island

## Abstract

**Objective:**

The arctic fox (*Vulpes lagopus*) is a circumpolar species inhabiting all accessible Arctic tundra habitats. The species forms a panmictic population over areas connected by sea ice, but recently, kin clustering and population differentiation were detected even in regions where sea ice was present. The purpose of this study was to examine the genetic structure of a population in the High Arctic using a robust panel of highly polymorphic microsatellites.

**Results:**

We analyzed the genotypes of 210 individuals from Bylot Island, Nunavut, Canada, using 15 microsatellite loci. No pattern of isolation-by-distance was detected, but a spatial principal component analysis (sPCA) revealed the presence of genetic subdivisions. Overall, the sPCA revealed two spatially distinct genetic clusters corresponding to the northern and southern parts of the study area, plus another subdivision within each of these two clusters. The north–south genetic differentiation partly matched the distribution of a snow goose colony, which could reflect a preference for settling into familiar ecological environments. Secondary clusters may result from higher-order social structures (neighbourhoods) that use landscape features to delimit their borders. The cryptic genetic subdivisions found in our population may highlight ecological processes deserving further investigations in arctic foxes at larger, regional spatial scales.

**Electronic supplementary material:**

The online version of this article (10.1186/s13104-017-3002-1) contains supplementary material, which is available to authorized users.

## Introduction

The arctic fox *Vulpes lagopus* has a circumpolar distribution and inhabits all accessible Arctic tundra habitats [1]. The species is relatively common across its range, except in Fennoscandia and on islands in the Bering Sea where populations are at critically low levels [1]. At the circumpolar scale, panmixia was reported in arctic foxes, due to the combination of connectivity offered by the sea ice and high mobility of this species [2–5]. Recently, however, kin clustering linked to female philopatry and fine-scale population genetic differentiation were found in Svalbard and Alaska despite the presence of sea ice in these regions [6, 7]. Currently, most studies using microsatellites in wild arctic fox populations typically used 10 markers or less [reviewed in 8]. Genetic homogeneity of populations was found at continental or circumpolar scales, but the use of a higher genetic resolution may allow detection of fine-scale genetic structure at more regional scales [9, 10].

Building on a previous study of arctic fox extra-pair mating in the same study area [11], we developed a panel of 15 microsatellite markers combined and amplified in two multiplex and one singleplex PCR assays. We used this set of highly polymorphic microsatellite markers to assess the genetic structure of a High Arctic population in a heterogeneous landscape.

## Main text

### Methods

**Fig. 1:**
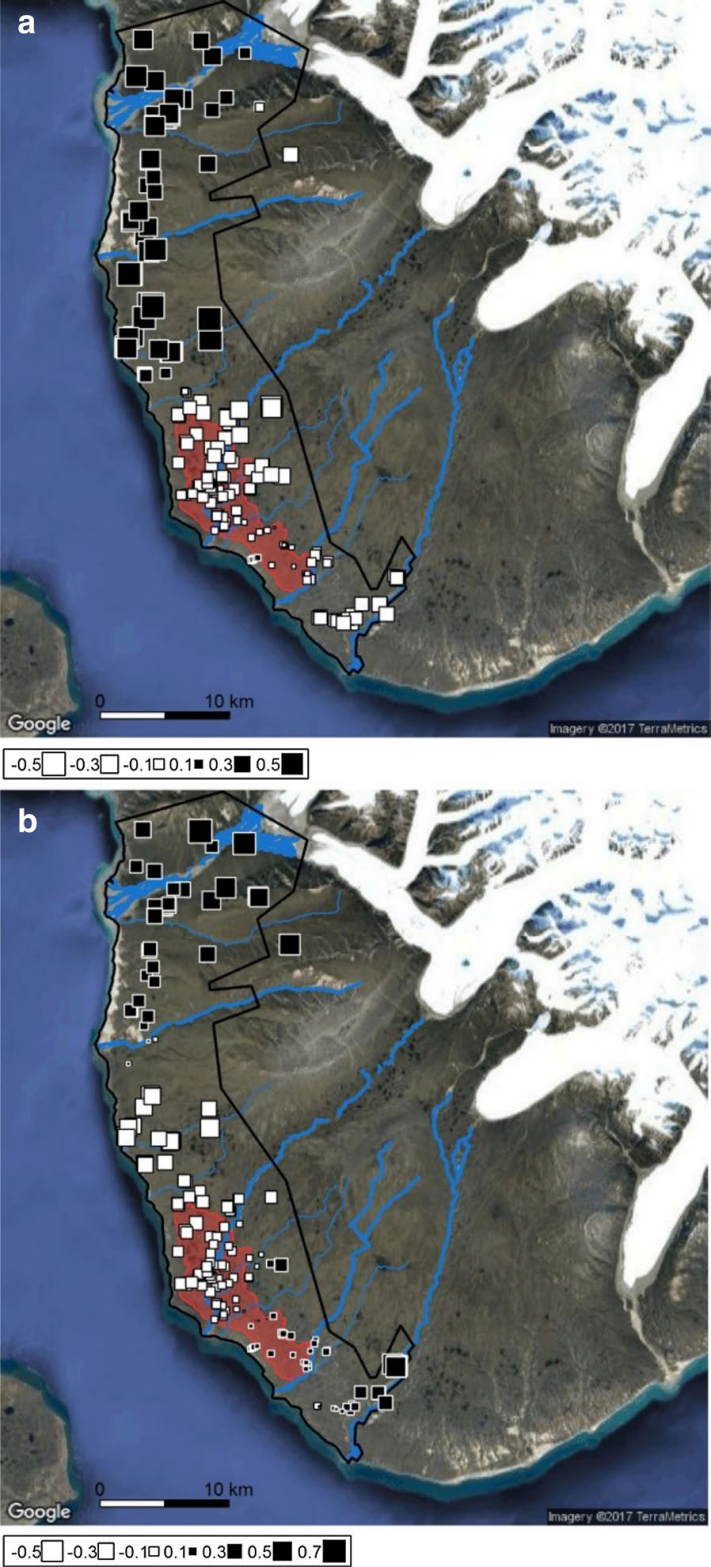
Map of spatial PCA global scores for arctic foxes in the south plain of Bylot Island (Nunavut, Canada) displaying **a** the first and **b** the second principal components. Each square represents the score of an individual positioned by its spatial coordinates. White squares show negative scores and black squares show positive scores. Larger squares reflect greater absolute values. Squares of different colors are strongly differentiated, while squares of the same color but of different sizes are weakly differentiated. The study area is delimited by a black line. Blue lines indicate rivers. The extent of a snow goose nesting colony is shown in red. Base map source from Google Maps: Imagery ©2017 Google, TerraMetrics

Tissue samples were collected from individuals captured on Bylot Island (73°N, 80°W), Nunavut, Canada, from 2003 to 2015, as detailed in Ref. [11]. The 600-km^2^ study area comprises approximately 60 km of coastline extending 5–15 km inland. The southern part of the study area hosts a Greater snow goose *Chen caerulescens atlantica* nesting colony during summer (Fig. 1). Total genomic DNA was extracted from ethanol-fixed ear samples stored at − 20 °C using the Qiagen DNeasy Kit. We randomly selected 15 samples from the tissue collection and 15 polymorphic primer pairs (Ref. [12–15]; Table 1) for microsatellite genotyping. These primers were used to determine optimal amplification conditions. PCRs were performed in a volume of 10 µl reaction containing 1.00 mM MgCl_2_, 1.00 µl DMSO, 1.00 µl BSA, 0.06 µl QBioTaq (all from Qiagen, Hilden, Germany), 0.40 µM dNTP (MP Biomedicals Europe, Illkirch, France), 0.25 µM each of forward and reverse primers (Eurofins Genomics, Ebersberg, Germany), 4.03 µl H_2_O and 2 ng DNA. Amplification conditions were as follows: 95 °C for 5 min, 30 cycles at 95 °C for 30 s, locus-specific annealing temperature (T_a_) for 1.3 min, 72 °C for 30 s and a final extension at 60 °C for 10 min. T_a_ of every primer pair was determined with a Mastercycler gradient 107 thermocycler (Eppendorf) PCR machine set according to the melting temperature (T_m_) values of primer pairs. The PCR products were electrophoresed on 6% denaturing polyacrylamide gels. These 15 primer pairs were selected to synthesize fluorescently-labeled primers for multiplex PCR.

**Table 1:** Summary information for the 15 microsatellite markers used in this study of *Vulpes lagopus*

Locus	Primer sequences (5′ to 3′)	Repeat motif	Size range (bp)	Number of alleles	Ho (He)	PCR multiplex	T_a_ (°C)	*F*_*IS*_	*F*_*null*_
CPH5 [12]	F:FAM-TCC ATA ACA AGA CCC CAA AC	(TG)_17_	84–112	14	0.788 (0.845)	1	57.4	0.070	0.038
	R:GGA GGT AGG GGT CAA AAG TT								
CPH9 [12]	F:PET-CAG AGA CTG CCA CTT TAA ACA CAC	(GT)_18_	152–164	6	0.671 (0.667)	1	57.4	− 0.004	0.001
	R:AAA GTT CTC AAA TAC CAT TGT GTT ACA								
CPH15 [12]	F:FAM-GCC TAT ATA AAA TGC ATC TGA GC	(AC)_18_	111–139	14	0.876 (0.890)	1	57.4	0.017*	0.015
	R:CCG TGA CTC CTG TCT TCT GAC								
CXX147 [13]	F:NED-CCA TGG GAA ACC ACT TGC	(AC)_20_	168–188	11	0.852 (0.842)	1	57.4	− 0.010*	0.009
	R:ACT TCA TCA TGT CTG GAA GCG								
CXX173 [13]	F:NED-ATC CAG GTC TGG AAT ACC CC	(TG)_17_	124–132	5	0.667 (0.739)	1	57.4	0.100	0.039
	R:TCC TTT GAA TTA GCA CTT GGC								
CXX250 [13]	F:PET-TTA GTT AAC CCA GCT CCC CCA	(AC)_18_A_2_(TC)_4_	120–140	11	0.862 (0.862)	1	57.4	0.007	0.009
	R:TCA CCC TGT TAG CTG CTC AA								
CXX733 [15]	F:VIC-CCCTCTACTTATGTCTCGGCC	Dinucleotide	245–271	12	0.576 (0.706)	1	57.4	0.187*	0.090
	R:GAGAGGAGAAACAACCAACACC								
CXX758 [15]	F:NED-AAG CAT CCA GAA TCC CTG G	Dinucleotide	213–253	17	0.857 (0.872)	1	57.4	0.019	0.020
	R:GTT GAT TGG GAG ATA ATC CAC A								
CXX771 [15]	F:VIC-GAG GAA GCC TAT GGT AGC CA	Dinucleotide	85–123	17	0.824 (0.851)	1	57.4	0.034*	0.018
	R:CAA GAC CTG AAT TCC TTG TTC C								
CPH3 [12]	F:NED-CAG GTT CAA ATG ATG TTT TCA G	(GA)_2_TA(GA)_17_	156–188	16	0.886 (0.888)	2	49.9	0.005	0.010
	R:TTG ACT GAA GGA GAT GTG GTA A								
CXX20 [13]	F:PET-AGC AAC CCC TCC CAT TTA CT	(CA)_21_	121–139	10	0.823 (0.848)	2	49.9	0.031	0.003
	R:TTG ATC TGA ATA GTC CTC TGC G								
CXX140 [13]	F:FAM-CAG AGG TGG CAT AGG GTG AT	(GT)_15_	137–161	11	0.790 (0.815)	2	49.9	0.033	0.009
	R:TCG AAG CCC AGA GAA TGA CT								
CXX745 [15]	F:FAM-TTT ATG GTC TCC ATG AGG GC	Dinucleotide	267–283	9	0.866 (0.850)	2	49.9	− 0.016	0.000
	R:TCC CTG CAT TTC CTT ATT TCA								
CXX671 [14]	F:VIC-AAA ATG AAA AAG GAA GAG AGC	(CA)_10_	199–215	9	0.880 (0.840)	2	49.9	− 0.044	0.000
	R:AGG AGA CAG GAT TTT CCT CTC A								
CXX377 [14]	F:VIC-ACG TGT TGA TGT ACA TTC CTG	(AC)_12_	173–193	11	0.843 (0.863)	Singleplex	58	0.026	0.016
	R:CCA CCC AGT CAC ACA ATC AG								

Size range, number of alleles, observed heterozygosity (Ho), expected heterozygosity (He), annealing temperature (T_a_), inbreeding coefficient (F_IS_) and null allele frequency (F_null_) are provided for each locus

* Significant deviation from Hardy–Weinberg equilibrium after Bonferroni correction

A Qiagen multiplex PCR Kit was used and reaction mixtures contained 1 µl DNA, 6.25 µl Master Mix (Qiagen, Hilden, Germany) with 1.25 µl primer mix (100 pM/µl) and 3.5 µl RNAse-free water to a final volume of 12 µl. The reaction comprised an activation step at 95 °C for 5 min, followed by 35 cycles of initial denaturation at 95 °C for 30 s, T_a_ for 30 s (Table 1) and 72 °C for 30 s, ending with a final extension step at 60 °C for 30 min, and followed by a holding step at 20 °C. Fluorescently-labeled PCR products were run on an ABI PRISM 3130 DNA Sequencer (Applied Biosystems) with the GeneScan-500 (LIZ) internal size standard and analyzed with the GeneMapper software (Applied Biosystems).

Fifteen loci (Multiplex_1_: 9 loci; Multiplex_2_: 5 loci; Singleplex: 1 locus; Table 1) were successfully amplified and genotyped in 210 specimens. We used R package *adegenet_2.0.1* [16] to estimate the number of alleles (NA), gene diversity He (expected heterozygosity), and observed heterozygosity (Ho) at each locus (Table 1). We used GENEPOP 4.1.3 [17] with a Markov chain method (10,000 dememorization steps, 100 batches and 5000 iterations per batch) to calculate inbreeding coefficients (F_IS_) and test for Hardy–Weinberg equilibrium (HWE) and linkage disequilibrium. Significance levels were adjusted for multiple tests using the sequential Bonferroni technique [18]. We used ML-NULLFREQ to estimate null allele frequencies based on maximum likelihood methods [19].

We investigated the fine-scale genetic structure using a global spatial autocorrelation technique (Mantel correlograms) [20, 21] implemented in the software GenAlEx 6.5 [22]. We used data of genotyped individuals that were resident in the study area (seven individuals dispersed, therefore n = 203). Spatial coordinates corresponded to occupied dens or capture sites. We used even distance classes of 4 km since this distance represents the mean radius of an arctic fox’s home range [23]. Statistical testing was based on 999 random permutations of individual genotypes. The complete dataset was first used, and then divided by sex (n = 99 males and 104 females) to test for sex-biased kin clustering. In addition, we used a spatially explicit multivariate method, the spatial principal component analysis (sPCA) implemented in *adegenet_2.0.1*. The sPCA takes into account both the genetic diversity based on allele frequencies (variance) and the spatial autocorrelation between individuals (Moran’s *I*). Global structures, such as distinct patches, genetic clines or intermediate states, appear when immediate neighbors are more genetically similar than expected under a random distribution. Local structures detect strong genetic differences among immediate neighbors, which could arise when individuals with a similar genetic pool avoid mating with each other. This approach does not require genetic equilibrium conditions and is useful for revealing cryptic spatial patterns [24], as demonstrated in other mammalian carnivores [20, 21, 25, 26]. We used a distance-based connection network, with a threshold distance between any two neighbors chosen as the minimum distance so that no individual was excluded from the graph [24]. As individuals could be associated to the same den, we added 100 m (in a random direction) to individual coordinates. We tested for significant global and local structure using a Monte-Carlo randomization test (999 permutations). We used the complete dataset first (threshold distance = 4359 m), and then the sex-specific datasets (threshold distance = 4766 m for males and 4424 m for females). For comparison, we also performed a Bayesian cluster analysis using the software STRUCTURE 2.3.4 [27].

### Results and discussion

The number of alleles per locus varied from five to 17. Expected heterozygosity ranged from 0.667 to 0.890, while observed heterozygosity ranged from 0.576 to 0.886. Null alleles occurred at low frequency (0.000–0.020) for 12 loci and at medium frequency for three loci (0.038–0.090). Significant departures from Hardy–Weinberg equilibrium after Bonferroni correction were detected at four loci (Table 1). Eight pairs of loci (7.6%) deviated significantly from linkage equilibrium after Bonferroni correction, a number only slightly higher than what may be expected by chance alone (5.25). As these deviations were very likely the result of the significant spatial genetic structure detected in the subsequent analyses, we included all loci in the analyses.

No significant spatial genetic structure was detected by the Mantel correlogram using the complete dataset (Mantel *r* = − 0.023 to 0.003; all *p* > 0.066) or sex-specific datasets (males: Mantel *r* = − 0.032 to 0.016, all *p* > 0.097; females: Mantel *r* = − 0.026 to 0.005; all *p* > 0.112). These results contrast with those of Ehrich et al. [6], who reported an isolation-by-distance pattern and female kin clustering in Svalbard. The observed trend was however very weak in their study (R^2^ = 0.0007, *p* = 0.027) and the median distance reported between first-order relatives (35.7 km) was relatively high [6].

The Monte-Carlo tests from the sPCA using the complete dataset revealed the existence of at least one global pattern (observed value = 0.009, *p* = 0.003) but no local structure (observed value = 0.010, *p* = 0.445). The first two eigenvalues were retained after examination of the eigenvalues barplot and the sPCA screeplot (Additional file 1: Figure S1). The scores from the first principal component mainly showed a latitudinal differentiation in the study area, with the southern cluster matching closely the extent of the goose colony (Fig. 1a). The scores from the second principal component mainly separated the individuals in the northernmost and southernmost parts of the study area from the rest, with the two clusters in the northern part (north of the goose colony) showing a sharper boundary (larger squares) than in the south (Fig. 1b). The southern part of the study area (goose colony and south of the colony) presented a north–south cline (genotypes located in the middle of the distribution had less extreme scores, as depicted by the smaller squares), indicating more mixing between the individuals in southern part of the goose colony.

Such cryptic population genetic structure in areas where no distinct physical barriers to movement occur may reveal interesting ecological processes. For instance, the principal southern cluster which approximately matched the goose nesting colony may reflect habitat imprinting (also termed, natal habitat-biased dispersal), which is a preference for settling into familiar ecological environments [28, 29]. This behavior has been suggested for arctic foxes to explain why the “lemming ecotype”, which feeds preferentially on small rodents, seems less likely to settle in areas inhabited by the “coastal ecotype”, which relies on resources from sea bird colonies and the marine environment [9, 30]. Habitat imprinting based on prey types or habitat features leading to cryptic genetic subdivisions has been reported in other canids, such as coyotes *Canis latrans* [31, 32] and gray wolves *Canis lupus* [21]. The smaller subdivisions revealed by the second principal component axis may be more difficult to explain. Their boundaries coincide approximately with rivers bordered by steep hillsides (Fig. 1b), but it is unlikely that these imped fox movements as foxes are regularly observed swimming across rivers (personal observations). However, the most distinct clusters may represent higher-order social structures (neighbourhoods occupied by extended family members) that may have borders delimited by landscape features such as rivers. Such an interaction between landscape features and social cohesion may become apparent at fine scales, as seen in the central California coyote population [32]. While remaining territorial, breeding pairs may adopt a good-neighbor strategy by tolerating relatives as neighbours, as observed in Norway where close relatives sometimes engage in communal breeding [33]. The sPCAs with either males or females did not reveal any pattern (data not shown), indicating no sex-biased genetic structures. The Bayesian cluster analysis inferred K = 2 as the most likely number of clusters (Additional file 2: Figure S2a). The percentage of individuals with membership coefficients *q* > 0.7 was relatively low (61%), highlighting a high level of admixture. Overall, individuals assigned to Cluster I and with a mixed membership were distributed all over the study area, while 73% of those assigned to Cluster II were found in the goose colony area (Additional file 2: Figure S2b, c).

The influence of social relationships on natal dispersal and spacing patterns, however, requires further investigation. From 2003 to 2015, a total of 371 pups were tagged in the population [annual pup production tagged (mean ± SD): 47 ± 27%]. Fourteen of these were resighted as adults and only six bred in the study area. Although not all pups in the study area were tagged, this suggests a lack of natal philopatry (or high mortality).

### Conclusions

This study confirms the absence of an isolation-by-distance pattern but reveals cryptic genetic subdivisions in our population. As reported in other studies [21, 31], using linear distances to estimate canid dispersal patterns may be misleading, especially in heterogeneous landscapes, and spatially explicit multivariate methods such as the sPCA may be more appropriate. Our multiplex assay consisting of 15 polymorphic loci will enhance analytical power to conduct fine-scale genetic analyses for this highly studied and locally endangered species which is the focus of > 30 monitoring projects throughout the world [34]. A comprehensive understanding of the spatial distribution of genetic diversity will inform conservation efforts.

## Limitations

One limitation of our study is that the study area may have been too small to detect a clear pattern of isolation-by-distance for a mobile species. Considering the relatively large distances between close relatives reported in Svalbard [6], foxes in sea ice regions may not display strict philopatry (staying in or moving to a site adjacent to the natal home range) as it is observed in Scandinavia [35]. Although logistically difficult to perform, increasing the sampling area may allow us to capture philopatry at a larger scale.

## Additional files

**Additional file 1: Figure S1.** Barplot of spatial principal component analysis (sPCA) eigenvalues and screeplot displaying each eigenvalue according to its variance and spatial autocorrelation (Moran’s *I*) components. Graphical results of the sPCA analysis of arctic foxes (n = 203) from Bylot Island, Nunavut, Canada.

**Additional file 2: Figure S2.** Bayesian genetic structure analysis conducted with the software STRUCTURE. Graphical results of the Bayesian genetic structure analysis of arctic foxes (n = 203) from Bylot Island, Nunavut, Canada conducted with the software STRUCTURE.
