# Supplementary material for: Fine-scale population genetic structure of arctic foxes (Vulpes lagopus) in the High Arctic
Source: BMC Res Notes. 2017 Dec 1;10:663. doi: 10.1186/s13104-017-3002-1 (PMC5710073; doi:10.1186/s13104-017-3002-1)
Supplement: Supplementary file 1 — Additional file 1: Figure S1. Barplot of spatial principal component analysis (sPCA) eigenvalues and screeplot displaying each eigenvalue according to its variance and spatial autocorrelation (Moran’s I) components. Graphical results of the sPCA analysis of arctic foxes (n = 203) from Bylot Island, Nunavut, Canada. [file 13104_2017_3002_MOESM1_ESM.pdf]

Additional file 1

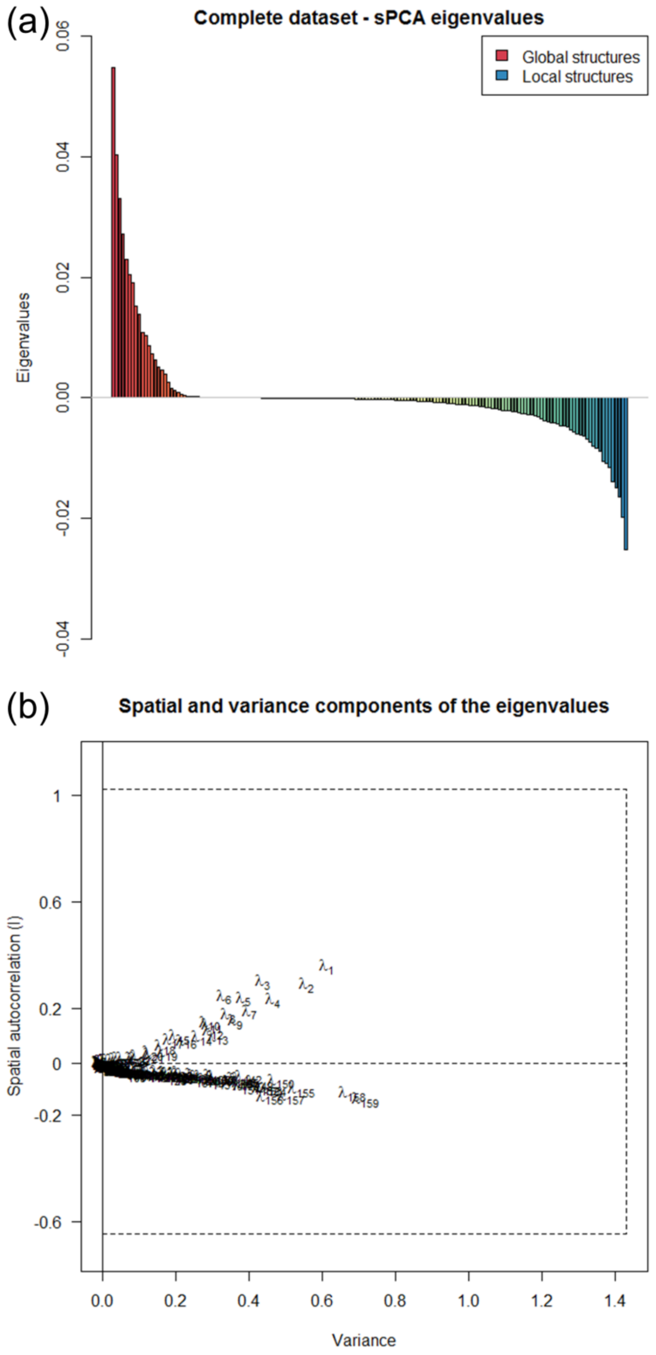

Figure S1. Barplot of spatial principal component analysis (sPCA) eigenvalues (a) and screeplot displaying each eigenvalue according to its variance and spatial autocorrelation (Moran's *I*) components (b). The two first global axes were retained for further analysis.
