# Supplementary material for: Fine-scale population genetic structure of arctic foxes (Vulpes lagopus) in the High Arctic
Source: BMC Res Notes. 2017 Dec 1;10:663. doi: 10.1186/s13104-017-3002-1 (PMC5710073; doi:10.1186/s13104-017-3002-1)
Supplement: Supplementary file 2 — Additional file 2: Figure S2. Bayesian genetic structure analysis conducted with the software STRUCTURE. Graphical results of the Bayesian genetic structure analysis of arctic foxes (n = 203) from Bylot Island, Nunavut, Canada conducted with the software STRUCTURE. [file 13104_2017_3002_MOESM2_ESM.pdf]

Additional file 2

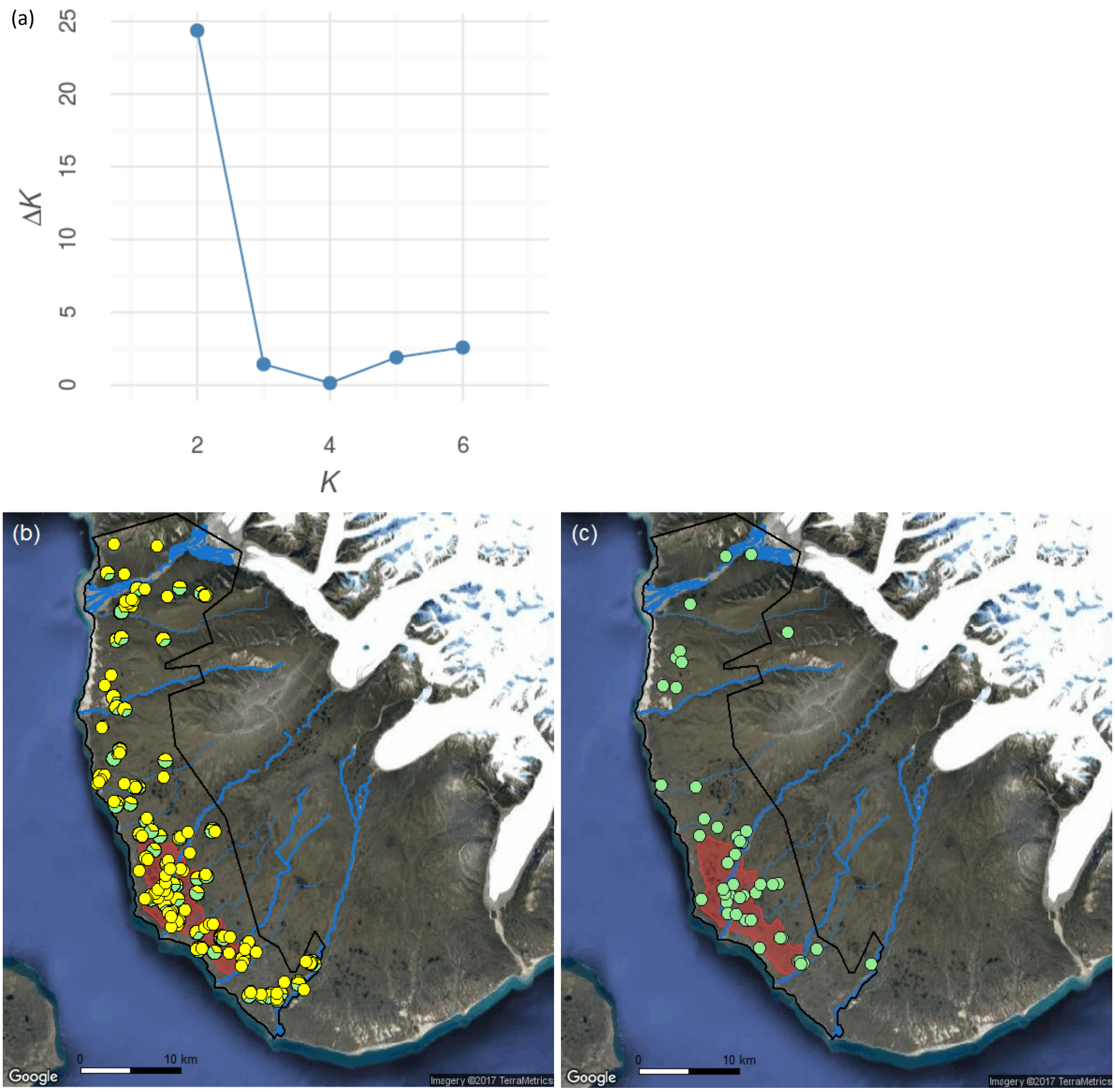

Figure S2. Results of the genetic structure analysis conducted with the software STRUCTURE. The Bayesian clustering analysis was performed using an admixture model with correlated allele frequencies, a number of clusters (K) between 1 and 7, and 100 000 burn-in steps followed by 1 000 000 MCMC replicates. Each value of K was replicated 10 times. (a) ΔK criterion of Evanno et al. (2005) used to determine the most likely number of genetic clusters (K = 2). (b-c) Map of the assignation of individuals (q > 0.7) to Cluster I (yellow; n = 79) and with a mixed membership (piecharts displaying the proportion of genome assigned to Cluster I and II; n = 80) and to Cluster II (green; n = 44). The study area is delimited by a black line. Blue lines indicate rivers. The extent of a snow goose nesting colony is shown in red. Base map source from Google Maps: Imagery ©2017 Google, TerraMetrics.

Reference

Evanno G, Regnaut S, Goudet, J. Detecting the number of clusters of individuals using the software STRUCTURE: A simulation study. Mol Ecol. 2005; 14; 2611-2620.
